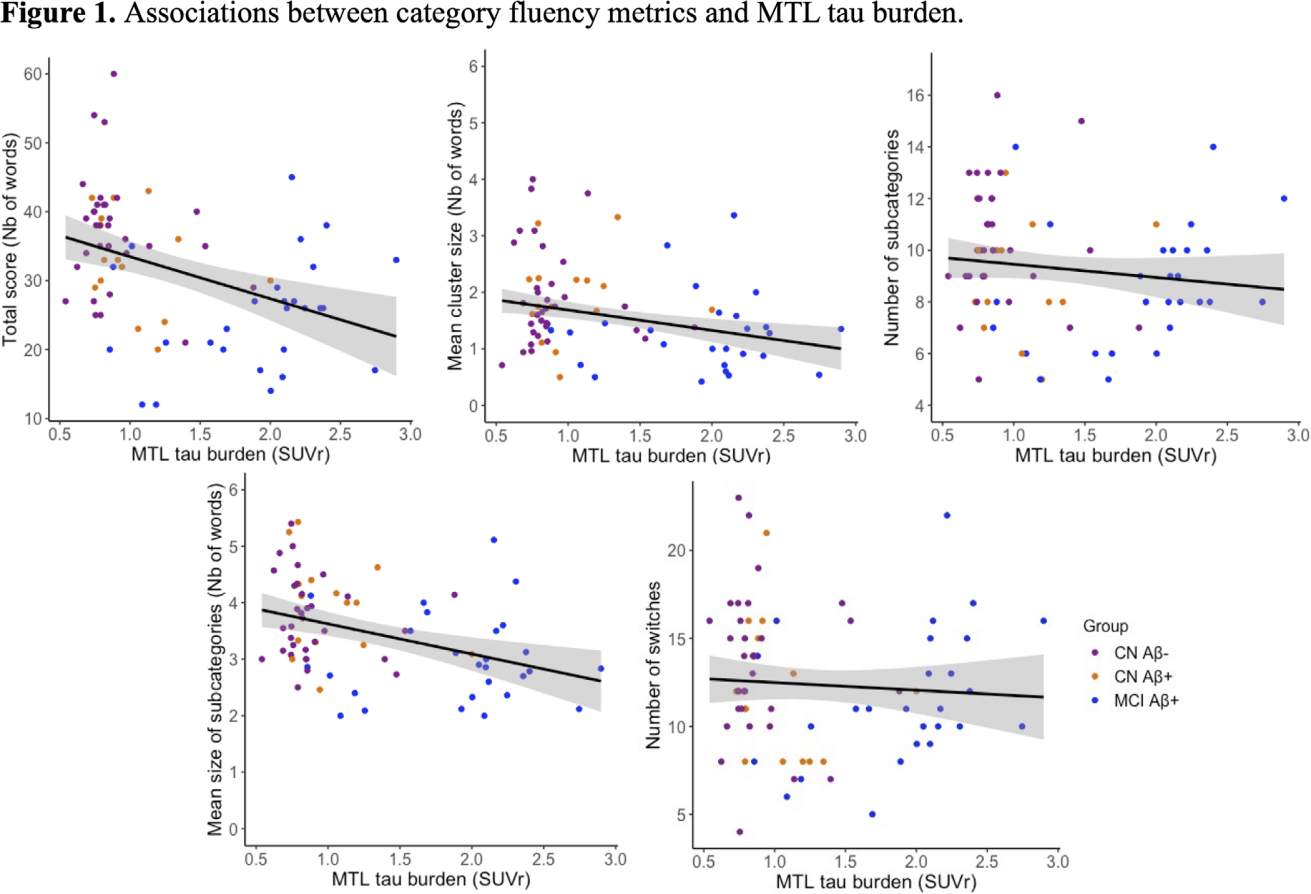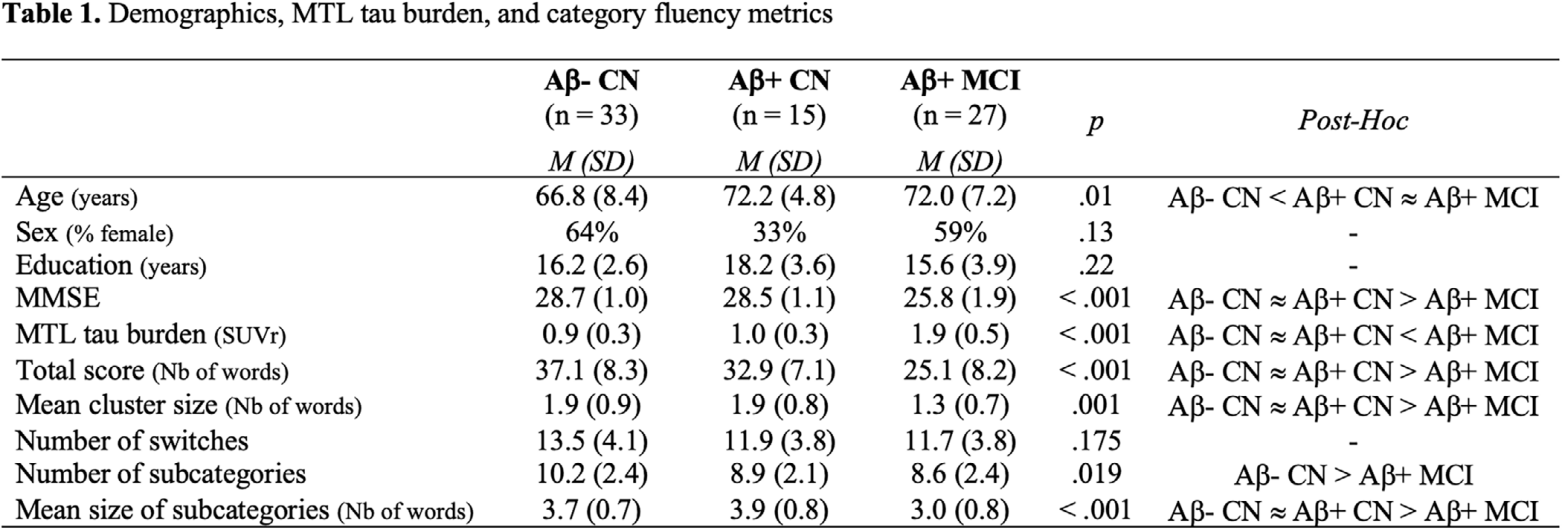# Associations between deep category fluency phenotyping and early tau aggregation in the medial temporal lobe

**DOI:** 10.1002/alz.095163

**Published:** 2025-01-09

**Authors:** Lisa Quenon, Emmanuelle Leroy, Mélanie Le Clec'H, Eva Scemama, Lara Huyghe, Lise Colmant, Yasmine Salman, Thomas Gérard, Vincent Malotaux, Laurence Dricot, Renaud Lhommel, Adrian Ivanoiu, Bernard J Hanseeuw

**Affiliations:** ^1^ Neuropsychological Rehabilitation Center, Saint‐Luc University Hospital, Brussels Belgium; ^2^ Institute of Neuroscience, UCLouvain, Brussels Belgium; ^3^ Faculty of Psychology and Educational Sciences, UCLouvain, Louvain‐la‐Neuve Belgium; ^4^ Massachusetts General Hospital, Harvard Medical School, Boston, MA USA; ^5^ Nuclear Medicine Department, Saint‐Luc University Hospital, Brussels Belgium; ^6^ Department of Neurology, Saint‐Luc University Hospital, Brussels Belgium; ^7^ Institute of Neuroscience ‐ UCLouvain, Brussels Belgium

## Abstract

**Background:**

Semantic processing relies on temporal brain regions, including medial temporal lobe (MTL) structures, that are the first to be affected by tau pathology in Alzheimer’s disease (AD). A widely used task to assess semantic memory is the category fluency. Performance on this task was demonstrated to be impaired since the prodromal stage of AD and associated to the structural integrity of the MTL. However, the associations between category fluency measures and MTL tau burden have been rarely explored at the early stages of the disease.

**Method:**

Seventy‐five participants underwent [^18^F]MK6240 tau‐PET, magnetic resonance imaging and cognitive assessment including category fluency (animals, 2 minutes). Amyloid‐b (Ab) status was determined with lumbar punction or [^18^F]Flutemetamol Aβ‐PET (Ab+ for cerebrospinal fluid Ab level<437pg/ml or Centiloid≥20). Participants included 33 Ab‐ cognitively normal (CN), 15 Ab+ CN and 27 Ab+ MCI individuals (Table 1). In addition to the category fluency total score, the mean cluster size (cluster = group of words that are sequentially produced and all contained within a subcategory such as birds), the number of subcategories evoked (aggregating semantically related clusters), the mean size of subcategories and the number of switches between clusters were calculated. Associations between these metrics and MTL tau burden were assessed using Spearman‐rank coefficients and generalized linear models adjusting for age, sex and education.

**Result:**

Over the entire sample, associations were found between the MTL tau burden and the total score (*r_s_* = ‐0.44, *p*<.001, Figure 1), the mean cluster size (*r_s_* = ‐0.29, *p* = .047), and the mean size of subcategories (*r_s_* = ‐0.40, *p* = .002), even when controlling for age, sex and education (b = ‐5.06, *SE* = 1.62, *p* = .003; b = ‐0.33, *SE* = 0.16, *p* = .039; b = ‐0.46, *SE* = 0.15, *p* = .003, respectively). The number of switches (*r_s_* = ‐0.13, *p* = 1.0) and the number of subcategories (*r_s_* = ‐0.13, *p* = 1.0) were not associated to the MTL tau burden.

**Conclusion:**

MTL tau burden was equivalently associated to the category fluency total score and two metrics relying more purely on semantic memory (mean size of clusters and subcategories). Given its critical role in conceptual knowledge and its involvement in the earliest stage of tauopathy, future work will analyze the associations between tau burden in the (peri)rhinal cortex and the category fluency metrics.